# Left orbital compartment syndrome and right anterior ischemic optic neuropathy in a patient with severe burns despite non-aggressive fluid resuscitation

**DOI:** 10.1177/20595131211006659

**Published:** 2021-04-14

**Authors:** Achmed Pircher, Sebastian Holm, Fredrik Huss

**Affiliations:** 1 Department of Neuroscience/Ophthalmology, Uppsala University, Uppsala, Sweden; 2 Burn Center, Department of Plastic- and Maxillofacial Surgery, Uppsala University Hospital, Uppsala, Sweden; 3 Department of Surgical Sciences, Plastic Surgery, Uppsala University, Uppsala, Sweden

**Keywords:** Burn, orbital compartment syndrome, ischemic optic neuropathy, fluid resuscitation, flame burns, lateral canthotomy

## Abstract

**Introduction:**

Ophthalmological complications such as orbital compartment syndrome (OCS) and ischemic optic neuropathy are rare complications in patients with burns and have been described in patients where aggressive fluid resuscitation was performed. While OCS requires urgent surgical intervention, no current treatment is established to treat, or prevent, ischemic optic neuropathy in patients with burns.

**Methods:**

The authors report a case of a 38-year-old woman with flame burns including the periorbital regions who developed OCS on the left side and anterior ischemic optic neuropathy (AION) on the right side despite non-aggressive fluid resuscitation. Immediate lateral canthotomy combined with inferior cantholysis was performed on the left side.

**Discussion and Conclusion:**

OCS and AION need to be considered as potential complications even in critically ill patients with facial burns who do not receive aggressive fluid resuscitation. Whether an early surgical intervention will lower the risk of AION development is, however, speculative.

**Lay Summary:**

Ophthalmological complications such as orbital compartment syndrome and ischemic optic neuropathy are rare complications in patients with burns and have been described in patients where aggressive fluid resuscitation was performed. We present a case of a critically ill patient with severe facial burns who developed orbital compartment syndrome on the left side and anterior ischemic optic neuropathy on the right side even though our patient did not receive aggressive fluid resuscitation.

Our case is particular because both of these rare complications are seen in a single patient and neither received aggressive fluid resuscitation. The fact that the patient did not develop ischemic optic neuropathy on the side where the lateral canthotomy was performed (only on the side where the patient had orbital compartment syndrome), this case might raise the discussion of whether an early surgical intervention might lower the risk of ischemic optic neuropathy development in patients with facial burns.

## Introduction

Orbital compartment syndrome (OCS) is characterised by an increase in intra-orbital pressure and requires quick surgical decompression to prevent permanent visual loss.^1^ The mechanism by which visual loss occurs in OCS is suggested to be an ischaemic process.^2^ As the orbit is confined by bony walls inferiorly, laterally, medially and to the anterior by a taut orbital septum and the eyelids, the orbit represents a compartmental structure. An increase in volume in such a compartment leads consequently to an increase in orbital pressure, which can indirectly be evaluated by measurements of elevated intraocular pressure (IOP).^3^ When the pressure is higher inside the orbit than within the arteries, the blood flow ceases, which leads to ischaemic damages to the optic nerve.^4^ The most common causes are intra-orbital haemorrhage secondary to trauma or surgical interventions. In the context of facial flame burns, OCS has been reported as a complication in patients where aggressive fluid resuscitation was performed.^5^

Ischemic optic neuropathy (ION) derives from ischemia in the optic nerve. It can be classified according to the anatomical localisation into an anterior ION (AION) and a posterior ION (PION). It is further classified into arteritic, caused by giant cell arteritis, and non-arteritic ION. ION is also a rare complication in non-ocular interventions such as spinal, cardiac, head, neck and general surgery.^6^ It has also been reported as a rare complication in major traumas that required resuscitation as a result of systemic hypotension secondary to sepsis.^7^ There are only very few case reports that described ION in association with burns.^8,9^ All of the reported patients suffered from major burns (>50% total body surface area [TBSA]) and all of them required initial aggressive volume fluid resuscitation.

This case presents a 38-year-old critically ill woman with severe flame burns, including the face and periorbital regions, who developed OCS on the left side and AION on the right side despite non-aggressive fluid resuscitation. The fact that lateral canthotomy was only performed on the left side raises the question of whether an early surgical intervention would lower the risk of ION development in critically ill patients with severe burns.

This case report followed the tenets of the Declaration of Helsinki. Consent was obtained from the patient for the use and publication of the patient’s case and photographs premortem.

## Methods

A 38-year-old woman was referred to our burn centre immediately after stabilisation according to the Advanced Trauma Life Support principles due to severe flame burns. The burn extent was eventually assessed to 80% TBSA including the face, trunk, and upper and lower extremities. Analgesia, mechanical ventilation after intubation and sedation were initiated. The burns in the face were full-thickness and included the periorbital region slightly more distinct on the left side. No facial fractures or injuries to the eye globe were present. Bronchoscopy revealed no signs of inhalation injury. The patient required immediate inotropic support.

Immediate fluid resuscitation was started and escharotomies were made on the abdomen and upper and lower extremities, and the patient was placed in a 30° recumbent position. The Parkland formula (volume of Ringer’s acetate = (2–) 4 mL × % TBSA × weight (kg)) was used for the calculation of the amount of fluid resuscitation for the first 24 h. The patient’s weight was 92 kg and the Parkland formula thus gives 29,400 mL to be infused within the first 24 h. However, the patient received only 19,200 mL Ringer’s acetate during the first 24 h (i.e. 3.5 mL × % TBSA × weight) because of an erroneous initial assessment of burn extent to only 60% TBSA. The urinary output was approximately 0.5 mL/kg/h, thus she was receiving adequate resuscitation.

Ophthalmological bedside examination about 24 h after the burn injury showed a swelling of the upper and lower eyelids with tautness on the left side. Marked chemosis of both the tarsal and bulbar conjunctivae was present on the left side and slightly on the right side; the corneal epithelium was unaffected bilaterally. The pupils were in miosis (pinpoint) and isocoric.

**Figure 1.**
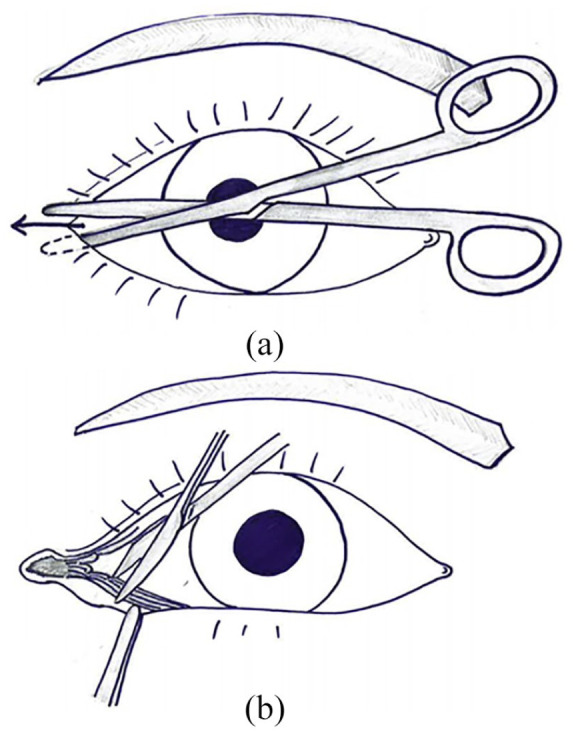
Illustration of lateral canthotomy and inferior cantholysis of the right eye. (a) Incision through the lateral canthus and canthal tenons. (b) Incision through the inferior lateral canthal tendon.

Repeated ophthalmological examination, 48 h after burn injury, revealed a marked increase of swelling and tautness of the upper and lower eyelids, mostly on the left side. Proptosis measurements were not reliable due to the severe swelling of the eyelids. The status of the pupils was unchanged compared to the previous examination and no relative afferent pupillary defect (RAPD) was present. The IOP measured with Icare Tonometry (Icare^®^, Vantaa, Finland) was 20 mmHg on the right side and 34 mmHg on the left side. Due to the increased and marked swelling and tautness of the eyelids in combination with elevated IOP, the clinical diagnosis of OCS on the left side was made and lateral canthotomy combined with inferior cantholysis was performed immediately (Figure 1). The IOP dropped immediately to around 15 mmHg after intervention. Over the following days, the swelling and tautness of the upper and lower eyelids continued to decrease on both sides. The general condition of the patient, however, remained critical and there were no signs of progressing wound healing.

**Figure 2.**
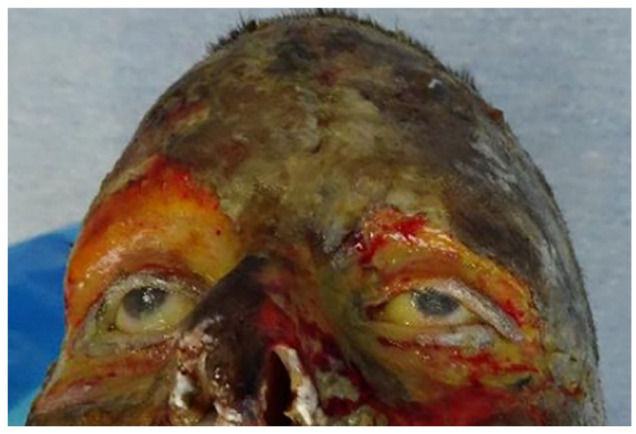
Photography of the upper part of the face of the patient in the case report. Note: Full-thickness burns including the periorbital regions. The image is copyright free, the consent has been obtained to use this image for publication.

**Figure 3.**
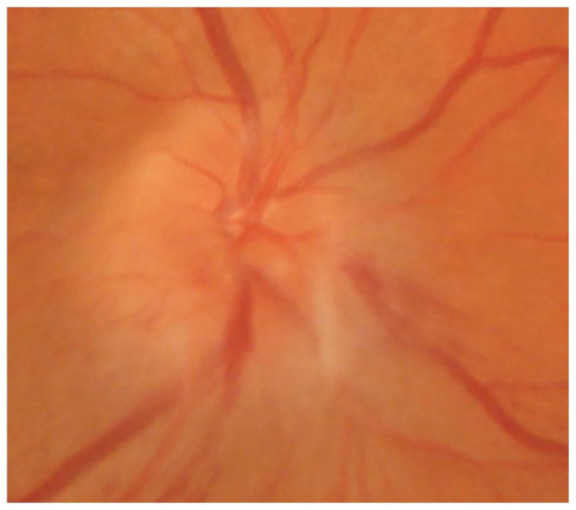
Fundus picture of a non-arteritic anterior ischemic optic neuropathy, similar to what was seen in the patient in the case report. Note: The image is copyright free, the consent has been obtained to use this image for publication.

On day 6 after the burn injury, the patient regained consciousness for the first time and described loss of vision in her right eye (by this time informed consent was obtained). Ophthalmologic examination showed almost fully regressed eyelid swelling with only a little conjunctival chemosis left and a clear cornea (Figure 2). Bilateral IOP measured at 12 mmHg. There was, however, no light perception on the right side and a RAPD was present. Fundoscopy showed a swollen optic disc with single disc haemorrhages (Figure 3). The optic disc on the left side was unaffected and had a normal appearance (normal C/D ratio). The clinical diagnosis of AION on the right side was made.

A total of 27 days after the burn injury, the patient went into septic shock and died from multiple organ failure.

## Discussion and Conclusion

This case presents a critically ill 38-year-old woman with severe flame burns (80% TBSA) including the periorbital region who developed OCS on the left side and AION on the right side despite non-aggressive fluid resuscitation. To the best of the authors’ knowledge, this case reports for the first time both these rare complications in a single patient with burns who did not receive aggressive fluid resuscitation.

The pathophysiology of OCS in burns is thought to derive from the excessive leakage of fluid and proteins into the orbit due to endothelial damage combined with an inflammatory reaction.^10^ High-volume fluid resuscitation within the first 24 h and periocular burns were shown to be risk factors for the development of a vision-threatening OCS in patients with burns.^5,11^ In the current case, the patient did not receive aggressive fluid resuscitation (3.5 mL × % TBSA × weight) within the first 24 h since the burn extent was at the initial assessment undervalued. However, the burn extent of the patient was large (80%) and the burns severe (deep-dermal to full-thickness). It may be possible that the severity of the patient’s burns induced an extensive inflammatory reaction and endothelial damage, which led to extensive leakage into the orbits even without aggressive fluid resuscitation.

The abovementioned mechanism is a systemic process and is not limited to only one side of the orbit. However, the patient developed OCS only on the left side. One possible explanation could be that the periorbital region on the left side was slightly more affected by the burns. In a retrospective study in burn patients, Singh et al.^11^ identified periocular burns as a potentially independent factor in the development of severe OCS. Burns to the eyelid lead to oedema and often tautness as also seen in the patient in this case report. While eyelid oedema may cause external pressure to the orbit, a more rigid eyelid will not allow appropriate elastic expansion in response to increased orbital volume and consequently contribute to the development of the OCS.

The other interesting peculiarity in this case is the development of an AION on the right side where no OCS was present. In association with burns, ION is not well described, and in all of the reported cases the ION was bilateral, the patients suffered from major burns (>55% TBSA), and all required initial aggressive fluid resuscitation and extended pressor support.^8,9^ In the present case, the patient also suffered from major burns and required extended pressor support; however, the ION was unilateral and the patient did not receive initial aggressive fluid resuscitation.

The blood flow in the optic nerve is determined by the ratio ‘perfusion pressure/resistance to flow’.^12^ In burns, endothelial damage occurs that causes capillary leakage and leads subsequently to the formation of oedema. This results not only in a decreased perfusion pressure due to intravascular volume reduction, but also in an increase in the resistance to flow due to the formation of interstitial oedema. It is possible that on the right side the intra-orbital pressure (which corresponds to the flow resistance) was too high in relation to the perfusion pressure and contributed to the development of the AION. It is not known whether the urgent orbital decompression, which successfully lowered the intra-orbital pressure (IOP measured 12 mmHg after the procedure) on the left side, prevented the development of an AION on that side. However, the question arises as to whether a canthotomy should be ‘generously’ performed in critically ill patients with severe burns and systemic hypotension.

A specific IOP value that could be the clinical trigger for performing canthotomy does not exist. Recent studies suggested an IOP greater than 40 mmHg, especially in combination with a RAPD as an indication for performing canthotomy.^13^ We recommend considering a canthotomy in critically ill patients with facial burns as soon as there is clinical suspicion of OCS, especially when expanded pressure support is required and do not recommend waiting necessarily until the IOP is greater than 40 mmHg. In patients with severe facial burns, both the evaluation of RAPD and the IOP measurement are due to the severe swelling of the conjunctivae and eyelids and pinpoint pupils difficult and often not reliable.

In order to reduce the risk for the development of OCS in patients with full-thickness facial burns, we recommend placing the patient in an as high as possible (preferrable 45°) recumbent position (usually limited by the blood pressure) and to generously perform escharotomies around the neck to decrease the intra-orbital pressure and to improve the blood circulation in the orbit. The Parkland formula should be used according to the standard values to calculate the estimated volume of resuscitation fluid. However, the actual amount of fluids given is to be based on keeping the urinary output at 0.5 mL/kg/h and mean arterial pressure >55 mmHg (adults). The effectiveness of pharmacological agents in lowering pressure in the intra-orbital compartment has not been established.

This case indicates that OCS and AION need to be considered as potential complications even in critically ill patients with facial burns who do not receive aggressive fluid resuscitation. Such patients should require a thorough ophthalmological examination and follow-up in order to identify an OCS in time. Further studies are needed to evaluate whether an early surgical intervention will lower the risk of AION development in critically ill patients with facial burns.
